# Large Congenital Cutaneous Mastocytoma Presenting With Recurrent Persistent Blistering: A Case Report

**DOI:** 10.7759/cureus.50306

**Published:** 2023-12-11

**Authors:** Jeva Cernova, Majeeda Patel, Marcin Ligaj, Portia Goldsmith, Sasha Dhoat, Edel O'Toole, Ravinder Atkar

**Affiliations:** 1 Dermatology, Barts Health NHS Trust, London, GBR; 2 Histopathology, Barts Health NHS Trust, London, GBR

**Keywords:** blister, tryptase, mastocytoma, congenital mastocytoma, cutaneous mastocytoma, mastocytosis

## Abstract

Congenital cutaneous mastocytoma is an uncommon disorder characterized by abnormal proliferation of mast cells. It typically presents as a single, small, yellowish-brown plaque, and its diagnosis is generally facilitated by distinctive clinical features, including a positive Darrier's sign. This report presents a case of an unusually large, solitary congenital mastocytoma encompassing nearly the entire circumference of the calf, observed in a newborn boy of Bangladeshi origin. Measuring 13x6 cm, the lesion formed large bullae and subsequent erosions. The perplexing clinical appearance prompted a skin biopsy, revealing monomorphic CD117 (c-KIT) positive infiltration without significant cell pleomorphism, confirming the diagnosis of cutaneous mastocytoma. The patient underwent management with potent and very potent topical steroids, oral antihistamines, and non-adhesive dressings, remaining under long-term follow-up with secondary care dermatology. In reporting this case, our objective is to augment the existing scientific literature by providing additional evidence that cutaneous mastocytomas can display a spectrum of clinical presentations, as illustrated in this case.

## Introduction

Mastocytosis is a condition caused by overproliferation and accumulation of tissue mast cells, frequently associated with CD117 (c-KIT) proto-oncogene mutation. It is a rare condition, with a prevalence estimated to be nine per 100,000 in the general population [1], with a male-to-female ratio of 1.4 [2]. The WHO classification distinguishes between cutaneous mastocytosis, which is more prevalent in children, and systemic mastocytosis, which is extremely rare in children [3]. Cutaneous mastocytosis is further subdivided into four types: maculopapular cutaneous mastocytosis (also known as urticaria pigmentosa), cutaneous mastocytoma, diffuse cutaneous mastocytosis, and telangiectasia macularis eruptiva perstans. In this case, we describe a congenital cutaneous mastocytoma. About 23% of mastocytosis cases are congenital [2].

## Case presentation

**Figure 1:**
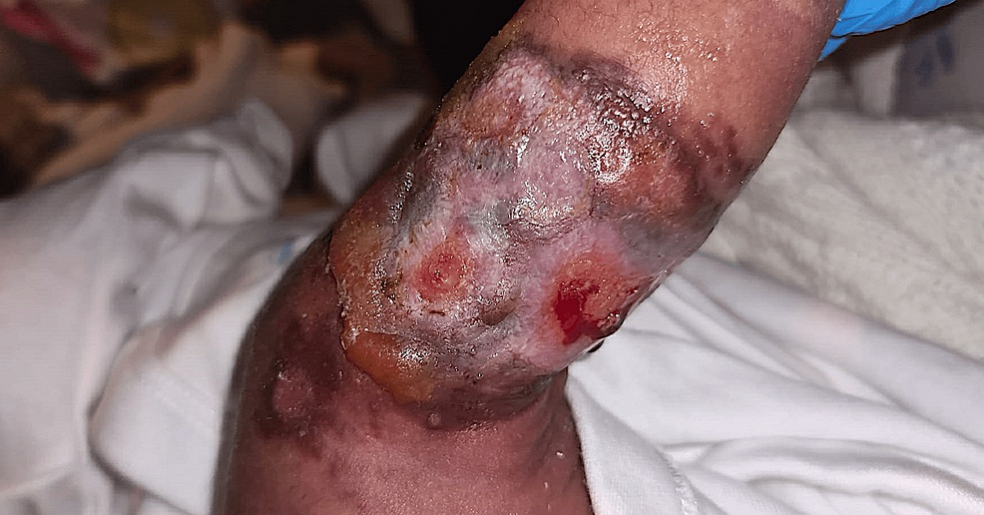
Erythematous and hyperpigmented plaque on the right calf, exhibiting oedema, bullae, and erosions.

A one-day-old Bangladeshi male presented with a congenital erythematous plaque with bullae on the right calf (Figure 1). He was born to non-related parents at term via cesarean section, and was systemically well. Pregnancy was complicated by gestational diabetes and polyhydramnios. The lesion measured 13 x 6 cm and covered almost the full circumference of the calf. Since birth, the lesion continued to change in appearance: swelled, blistered, ulcerated and healed. Darier's sign was positive. There was no hepatosplenomegaly or lymphadenopathy. 

**Figure 2:**
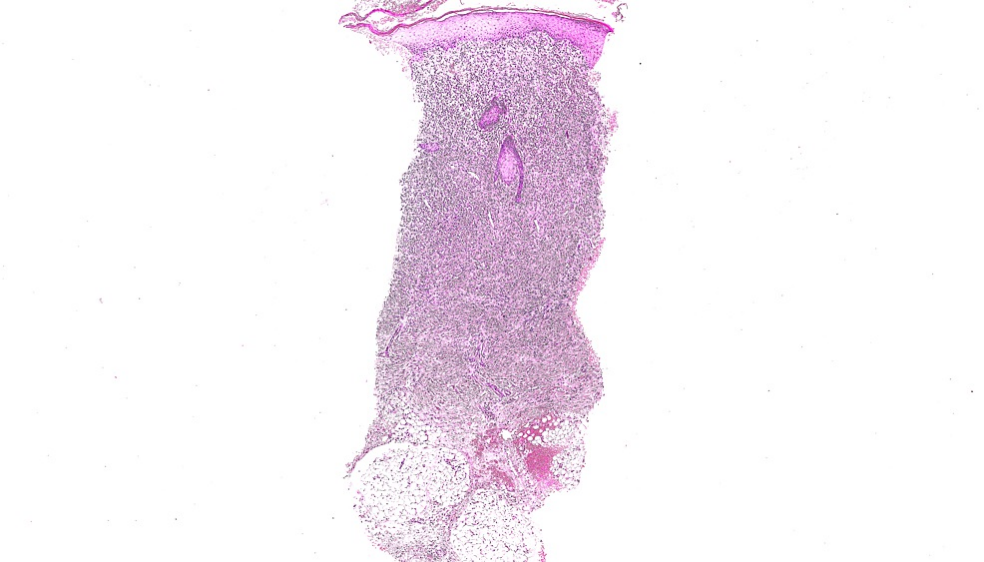
Haematoxylin and eosin staining of the punch biopsy (low power magnification) demonstrated full-thickness infiltration limited to the dermis and reaching down to subcutaneous tissue.

**Figure 3:**
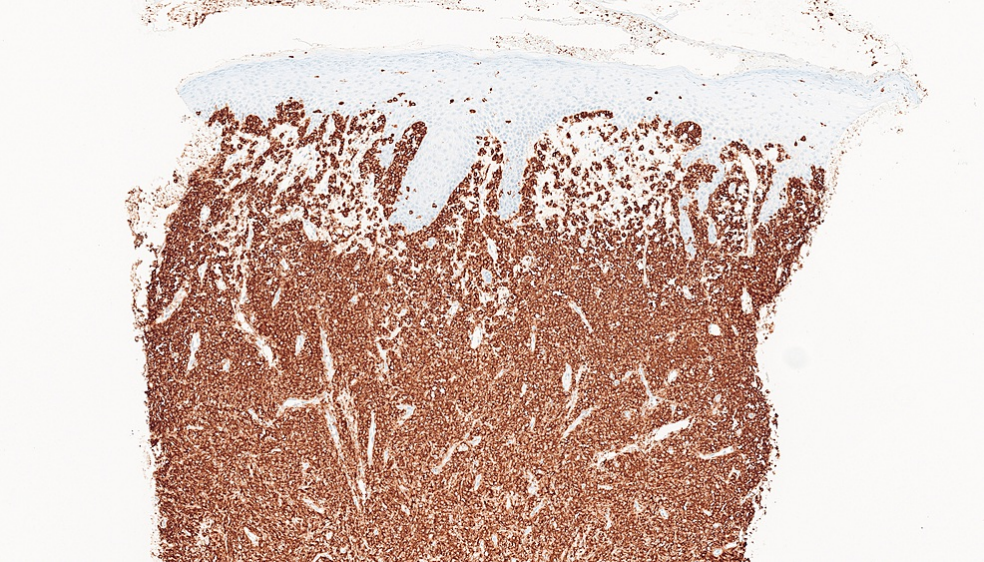
Biopsy with CD117 staining (medium power) showed the infiltrate diffusely and strongly positive for CD117, consistent with mast cell lineage

Routine biochemical and haematological investigations were within normal limits, and bacterial and viral cultures were negative. A diagnostic punch biopsy was performed from the plaque area, blistering site, and lesion edge. Histopathology revealed full-thickness dermal infiltration (Figure 2) with a dense monomorphic infiltrate of epithelioid and spindle cells without significant pleomorphism. Immunohistochemistry with anti-c-KIT was strongly positive (Figure 3). Staining with Ki-67 demonstrated low mitotic activity. Toluidine blue staining demonstrated metachromasia (the colour of the intracellular granules changed to purple) allowing the identification of mast cells. Further investigations showed a normal serum tryptase level of 6 micrograms/L. Serum tryptase is a marker of mast cell activation and degranulation.

Treatment was initiated with mometasone furoate 0.1% ointment once a day for two weeks, followed by alternate-day application. While initially effective, after four months there was an increased frequency of blistering. Treatment was escalated to once daily clobetasol propionate 0.05% ointment with good results. Topical steroid application was adjusted based on blistering occurrences. A non-adhesive dressing was used to reduce blistering from friction as the child became mobile. Antihistamines, starting with chlorpheniramine maleate 1 mg twice daily, were used. Four months later, cetirizine was added at 1.8 mg twice a day (0.25 mg/kg per dose) and increased to 2.5 mg three times a day (0.25mg/kg/dose) six months later when weight reached 11 kg. The lesion persists at 16 months of age with fewer and less severe blistering episodes. There are no features suggestive of systemic involvement and serum tryptase remains within normal limits. A six-monthly follow-up with annual serum tryptase measurements was advised. 

## Discussion

Cutaneous mastocytoma typically presents as a solitary plaque, with colours ranging from orange-yellow to reddish-brown and a diameter of 1-5 cm [1]. Over the past decade, a handful of case reports have documented cutaneous mastocytomas in the pediatric population. Notable instances include a report of 11 discrete mastocytomas occurring simultaneously in a male patient shortly after birth [4], and a similar case describing multiple blistering and eroding lesions initially suspected to be epidermolysis bullosa [5]. Additionally, cases underscore the potential for misdiagnosis, such as that of a two-month-old boy with a mastocytoma on the lower eyelid initially misdiagnosed as pre-septal cellulitis due to peri-orbital swelling and erythema [6].

Mastocytomas may exhibit systemic symptoms and manifest in various body parts, exemplified by a case of vulval mastocytoma presenting with recurrent urticaria, emphasizing the importance of comprehensive physical examination, including examination of genital skin [7]. Further case reports document unusual histological features, such as mastocytoma with features of granuloma faciale (such as grenz zone, dermal telangiectasia and mixed inflammatory infiltrate) [8], pseudo carcinomatous hyperplasia and scattered eosinophils [9], eosinophilic infiltrate and flame figures, resembling histological features of Wells syndrome [10], hypervascular pseudo angiomatous histopathology, and xanthelasmoid clinical appearance [11].

Overall, recurrent blistering and urtication at a single site is almost pathognomonic of a cutaneous mastocytoma. In the differential diagnosis, we considered a segmental form of diffuse cutaneous mastocytosis, aplasia cutis congenita, and localized epidermolysis bullosa. Other conditions to consider include infections (herpes simplex virus (HSV), bullous impetigo, staphylococcal scalded skin syndrome, congenital syphilis) trauma, and drug-related and autoimmune blistering conditions. 

In bullous impetigo, blisters are fragile and readily rupture, often showing a characteristic golden crust suggesting *Staphylococcus aureus *infection. HSV infection is unlikely to present as a single localized plaque. More recently, there have been reports of multisystem inflammatory syndrome (MIS-C) in children linked to severe acute respiratory syndrome coronavirus 2 (SARS-CoV-2), with cases featuring blistering rashes [12]. Chikungunya virus is primarily spread by mosquitoes but has also been reported with peripartum mother-to-child transmission. It can cause diffuse maculopapular rashes with bullae formation that typically starts on the lower limbs [13]. Neonates with these infections often have systemic symptoms. 

Aplasia cutis congenita can present as a well-demarcated atrophic plaque characterised by the absence of skin and, in rare cases, can initially present as a bulla, recognized as bullous aplasia cutis congenita [14]. Both epidermolysis bullosa and mastocytoma can form blisters due to friction; however, epidermolysis bullosa is typically widespread. Neonatal epidermolysis bullosa acquisita can occur in a newborn due to the transplacental transfer of pathogenic maternal IgG and typically follows a self-limiting course [15]. Neonatal lupus erythematosus can result in early-life blistering that heals with milia [16] and is important to recognise as it can result in congenital atrioventricular heart block with high morbidity and mortality.

A rare differential that can be considered in view of mast cell proliferation is mast cell sarcoma, an aggressive but exceedingly rare cancer with very few cases reported to date. It has been reported in an infant in whom it was associated with raised serum tryptase and histology demonstrated rounded mast cells with mild-to-moderate degrees of atypia, positive for CD117, with notable absence of metachromatic granules [17]. Another rare condition is Langerhans cell histiocytosis, a rare proliferative disease with potential for malignant transformation that can present with vesiculo-pustular or vesiculo-haemorrhagic lesions at birth [18]. 

The importance of early recognition of mastocytosis is emphasized by a case in which an infant had an extensive bullous reaction associated with anaphylactic shock following the administration of codeine for pain relief for mastocytosis misdiagnosed as bullous impetigo [19]. Mast cell degranulation can be provoked by various medications, including opioids, non-steroidal anti-inflammatory drugs (NSAIDs), certain anesthetic agents, antibiotics, and contrast dyes. The prevalence of anaphylaxis in paediatric patients with cutaneous mastocytosis is estimated at 5.5% [2]; however, there seems to be no positive correlation between tryptase levels and the risk of anaphylactic reaction [20]. 

In cutaneous mastocytosis, normal blood test results, low serum tryptase (<20 micrograms/L), absence of hepatomegaly, splenomegaly, lymphadenopathy, and systemic symptoms are strongly suggestive of the disease being limited to the skin. A serum tryptase level >20 micrograms/L is one of the diagnostic criteria for systemic mastocytosis [3]. This should be interpreted with caution in the pediatric population but could suggest a systemic process. It is estimated that approximately 3% of pediatric mastocytosis cases progress to mast cell lymphoma, mast cell sarcoma, or a systemic form [2]. 

Most cutaneous mastocytomas have a good prognosis with complete or partial involution in two-thirds of cases by adulthood [1,2]. Treatment is mainly supportive with emollients and H1-blocking antihistamines. A study comparing a “wait and see” approach versus treatment with a high-potency topical corticosteroid demonstrated improved resolution time in the corticosteroid group [21]. Further treatment options for cutaneous mastocytomas include phototherapy and topical calcineurin inhibitors. In cutaneous mastocytosis, systemic treatment is rarely required; however, in very severe cases, potential therapeutic strategies include omalizumab, tyrosine kinase inhibitors such as imatinib mesylate and mesatinib, cladribine, as well as topical agent miltefosine [22]. 

## Conclusions

We presented a rare case of a large congenital cutaneous mastocytoma in a newborn, measuring 13 cm by 6 cm, that posed diagnostic challenges due to its extensive size, protean appearance, and recurrent blistering. The diagnosis was confirmed through biopsy, revealing full-thickness dermal infiltration of mast cells. The management involved the use of topical steroids, oral antihistamines, and non-adhesive dressings. Despite the initial efficacy of mometasone furoate 0.1% ointment, treatment necessitated an escalation to clobetasol propionate 0.05% ointment, emphasizing the significance of ongoing monitoring and follow-up in secondary care. In view of the large size of the lesion, we do not expect complete resolution with time. In this report, we also described uncommon causes of blistering skin in newborns, emphasizing the need for accurate and timely diagnosis.
